# Ramsay Hunt Syndrome Associated with True Vocal Cord Palsy- A Case Report 

**Published:** 2019-03

**Authors:** Mohsen Rajati, Mohammad Ali Zarringhalam

**Affiliations:** 1 *Sinus and Surgical Endoscopic Research Center, Mashhad University of Medical Sciences, Mashhad, Iran. *; 2 *Departments of Otorhinolaryngology, Mashhad University of Medical Sciences, Mashhad, Iran.*

**Keywords:** Polyneuropathy, Ramsay Hunt syndrome, True vocal cord palsy

## Abstract

**Introduction:**

Varicella-zoster virus may cause an infectious disease called Ramsay Hunt syndrome. The related symptoms include facial nerve palsy (FNP), otalgia, the vesicular eruptions of the auricle and external auditory canal, less common ocular movement disorder, facial hypoesthesia, myofascial pain, vestibular symptoms, hearing loss, dysphasia, vocal cord paralysis, as well as tongue paralysis due to cranial neuropathies.

**Case Report:**

Herein, we presented the case of a 55-year-old man with left peripheral facial nerve palsy, profound hearing loss, and true vocal cord paralysis. The FNP recovered after 2 weeks and synkinesis totally improved after 4 weeks.

**Conclusion:**

Ramsay Hunt syndrome may present as cranial polyneuropathy; therefore, accurate history taking and physical examinations are necessary in this regard. The recovery rate of the vagus nerve is probably fair without polyneuropathy; however, it seems to be poor in cases suffering from polyneuropathy.

## Introduction

Ramsay Hunt syndrome (RHS) is an infectious disease caused by varicella-zoster virus (VZV). This syndrome is characterized by facial nerve palsy (FNP), otalgia, and the vesicular eruptions of the auricle, as well as external auditory canal.

With the incidence rate of 4.5%-9%, VZV is the most common cause of FNP after Bell’s palsy (1). 

The related incidence rate increases in patients over 60 years of ages. Cellular immunity is compromised in 10% of VZV-infected patients that may be due to cancer, trauma, radiotherapy, or chemotherapy (2). Other risk factors, which have been reported in the literature, are emotional stress, smoking, diabetes, depression, organ transplantation, and medication that suppress the immune system (3). 

Ramsay Hunt syndrome is considered to be more severe with poorer prognosis, in comparison with Bell’s palsy. Ramsay Hunt syndrome may be associated with other cranial neuropathies, including ocular movement disorder, facial hypoesthesia, myofascial pain, vestibular symptoms, hearing loss, dysphasia, and vocal cord paralysis, as well as tongue paralysis (3). 

In a study, 1.8% (11 out of 615 cases) of all reported Ramsay Hunt syndromes correlated with other cranial neuropathies (4). In the present report, a case of Ramsay Hunt syndrome with concomitant paralysis of nerves VII, VIII, and X was presented and the prognosis was discussed. 

## Case Report

We presented the case of a 55-year-old man with left peripheral facial nerve palsy. The symptoms appeared one week earlier, along with viral upper respiratory infection. 

**Fig 1:**
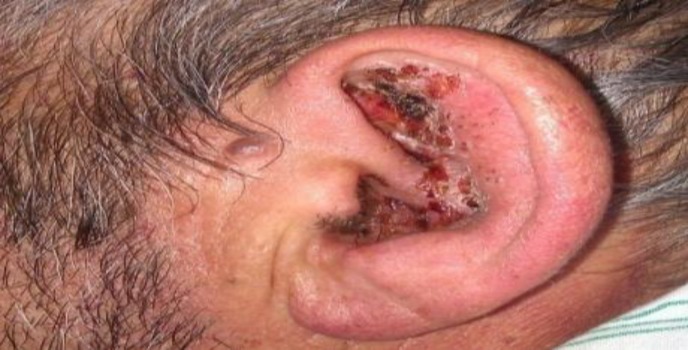
View of auricle

**Fig 2:**
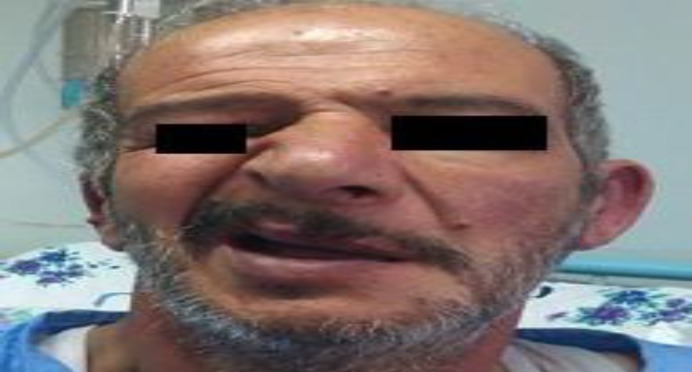
Peripheral facial nerve palsy

Otalgia started after two days accompanied by hearing loss and tinnitus on the left side; on the third day, vesiculoerosive lesions manifested itself in the left auricle (Fig.1), followed by the incidence of left side FNP (Fig.2).

The patient also suffered from true vertigo. On the same day of FNP incidence, the case developed hoarseness and fluid aspiration that became worse in the following days. The case had a surgery on the other ear (right side) 20 years earlier and 20 pack-year history of smoking. There were no diplopia, ptosis, headache, and the weakness of limbs or loss of consciousness. In addition, the patient had no history of diabetes or tuberculosis.

**Fig 3:**
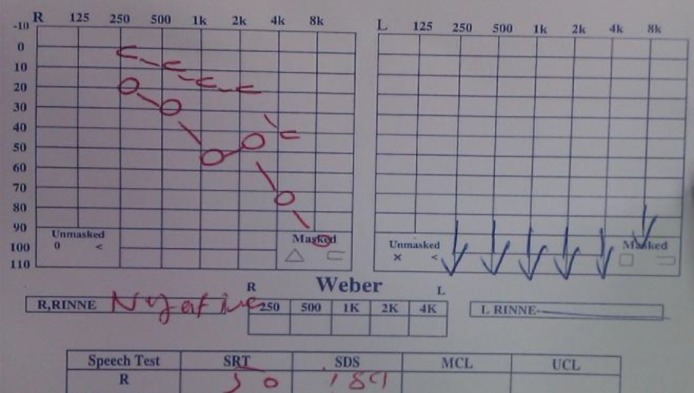
Audiometric assessment

The physical examination revealed a left peripheral FNP grade VI based on House-Brackmann (HB) grading. In the right ear, he had previous surgical scar, along with central perforation with otorrhea. In the left ear, herpetic vesicles, as well as papules and pustules, associated with erythema and edema were observed in the ear canal and the conchal bowl (Fig.1). The laryngoscopic examination confirmed left vocal cord paralysis that was fixed in the paramedian position. The audiological evaluation verified left side profound hearing loss (Fig.3). 

The patient was managed with RHS diagnosis associated with the paralysis of cranial nerves VIII and X, and the treatment started with acyclovir and prednisone 1mg⁄kg. 

The FNP recovered after 2 weeks and the synkinesis totally improved following 4 weeks. Although fluid aspiration remarkably improved, left recurrent laryngeal nerve paralysis persisted on indirect laryngoscopy, with the compensation from opposite vocal cord. The case’s vertigo improved after 4 days of treatment; however, occasional non-pulsatile tinnitus still lingered on. The vesicles changed into scabs within 5 days and the inflammation and erythema disappeared after 2 to 3 weeks. The left side sensorineural hearing loss and also recurrent laryngeal nerve paralysis sustained for a year of follow-up.

## Discussion

In a study carried out by Kim et al. (4), the most commonly involved cranial nerves in Ramsay Hunt syndrome associated with cranial polyneuropathy were VII, VIII, IX, X, V, III and XII, respectively; other researchers reported this involvement in a little different order as follows: VII, VIII, IX, V, X, and VI (5).The recovery rate of facial nerve paralysis in Ramsay Hunt syndrome is considered to be low (6). However, there are limited available data regarding facial paralysis prognosis in this syndrome when associated with polyneuropathy. In the study conducted by Kim et al. the rate of complete recovery (HB grade І) was reported as 45.5% and fair recovery (HB grade II and III) was 81.8% demonstrating that the recovery rate of FNP is not low in RHS associated with polyneuropathy (4).

Similarly, in the present case, medical treatment resulted in the complete recovery of facial nerve paralysis (HB grade I). In Kim et al. report (4), the cranial nerve VIII was the most commonly involved nerve (in 90.9% of the cases) following the facial nerve; however, the recovery rate was very low (11.1%). In the present case, it was also observed that after 12 months of follow-up, the hearing loss did not improve.

In a study carried out by Sun et al. (3), an atypical case of RHS was described in a diabetic patient that involved the cranial nerves V, VII, VIII, and XII. In the aforementioned study, the patient was satisfactorily recovered following 3 months of antiviral therapy, antibiotics, insulin therapy, and traditional Chinese drugs.

In a case study conducted by Arya RHS on an HIV-positive patient, only moderate improvement occurred in the ability to swallow (cranial nerve VII) after 10 days of medication therapy; nonetheless, in 120 days of follow-up, other impairments were continued (7).

Keishi Fujiwara et al. reported a case of zoster sine herpete with the paralysis of left vocal cord (8), left facial nerve palsy, and left soft palatal weakness. The neurological disorders of the patient completely improved with antiviral drugs and corticosteroids therapy.

A review study was carried out by Eva Rye Rasmussen et al. on RHS associated with the paralysis of the recurrent nerve since 1960 (9). The complete recovery rate was reported as 67.7%–82.9% in isolated RHS; however, RHS with multiple cranial nerves involvement decreased the recovery rate as low as 27.3%. Only the complete recovery rate of facial nerve paralysis was 45.5%–54.5%; nonetheless, the recovery rate of hearing loss was estimated as 11%–80%. The recovery rate for vocal cord paralysis seems to be better.

Kim et al. reported the complete recovery rate of vagal nerve paralysis as 60% (4). Other commonly involved cranial nerves in RHS are IX, X, and V all of which are reported to have fair recovery rates. In contrast, in the present case, vagal nerve palsy did not improve after 12 months of follow-up and left vocal cord paralysis persisted. However, aspiration and voice quality gradually improved due to the compensation of the other side.

## Conclusion

Ramsay Hunt syndrome may present as cranial polyneuropathy; therefore, accurate history taking and physical examinations are necessary. The recovery rate of the vagus nerve is probably fair without polyneuropathy; however, it seems to be poor in cases suffering from polyneuropathy. 
